# Prevalence of Allergic Reactions After Pfizer-BioNTech COVID-19 Vaccination Among Adults With High Allergy Risk

**DOI:** 10.1001/jamanetworkopen.2021.22255

**Published:** 2021-08-31

**Authors:** Ronen Shavit, Ramit Maoz-Segal, Mona Iancovici-Kidon, Irena Offengenden, Soad Haj Yahia, Diti Machnes Maayan, Yulia Lifshitz-Tunitsky, Stanley Niznik, Shirly Frizinsky, Michal Deutch, Eti Elbaz, Hosney Genaim, Galia Rahav, Itzchak Levy, Anna Belkin, Gili Regev-Yochay, Arnon Afek, Nancy Agmon-Levin

**Affiliations:** 1 Clinical Immunology, Angioedema and Allergy Unit, Center for Autoimmune Diseases, Sheba Medical Center, Tel Hashomer, Israel; 2 Sackler School of Medicine, Tel Aviv University, Tel Aviv, Israel; 3 Infectious Diseases Unit, Sheba Medical Center, Tel Hashomer, Israel; 4 Infection Prevention and Control Unit, Sheba Medical Center, Israel; 5 Sheba Medical Center, Tel Hashomer, Israel

## Abstract

**Importance:**

Allergic reactions among some individuals who received the Pfizer-BioNTech (BNT162b2) COVID-19 vaccine discourage patients with allergic conditions from receiving this vaccine and physicians from recommending the vaccine.

**Objective:**

To describe the assessment and immunization of highly allergic individuals with the BNT162b2 vaccine.

**Design, Setting, and Participants:**

In a prospective cohort study from December 27, 2020, to February 22, 2021, 8102 patients with allergies who applied to the COVID 19 vaccine referral center at the Sheba Medical Center underwent risk assessment using an algorithm that included a detailed questionnaire. High-risk patients (n = 429) were considered “highly allergic” and were immunized under medical supervision.

**Exposures:**

Pfizer-BioNTech (BNT162b2) COVID-19 vaccine.

**Main Outcomes and Measures:**

Allergic and anaphylactic reactions after the first and second doses of BNT162b2 vaccine among highly allergic patients.

**Results:**

Of the 429 individuals who applied to the COVID-19 referral center and were defined as highly allergic, 304 (70.9%) were women and the mean (SD) age was 52 (16) years. This highly allergic group was referred to receive immunization under medical supervision. After the first dose of the BNT162b2 vaccine, 420 patients (97.9%) had no immediate allergic event, 6 (1.4%) developed minor allergic responses, and 3 (0.7%) had anaphylactic reactions. During the study period, 218 highly allergic patients (50.8%) received the second BNT162b2 vaccine dose, of which 214 (98.2%) had no allergic reactions and 4 patients (1.8%) had minor allergic reactions. Other immediate and late reactions were comparable with those seen in the general population, except for delayed itch and skin eruption, which were more common among allergic patients.

**Conclusions and Relevance:**

The rate of allergic reactions to BNT162b2 vaccine, is higher among patients with allergies, particularly among a subgroup with a history of high-risk allergies. This study suggests that most patients with a history of allergic diseases and, particularly, highly allergic patients can be safely immunized by using an algorithm that can be implemented in different medical facilities and includes a referral center, a risk assessment questionnaire, and a setting for immunization under medical supervision of highly allergic patients. Further studies are required to define more specific risk factors for allergic reactions to the BNT162b2 vaccine.

## Key Points

**Question:**

Can patients at high risk for anaphylactic reactions receive the Pfizer-BioNTech (BNT162b2) COVID-19 vaccine?

**Findings:**

In this cohort study of 8102 individuals with an allergy history, an algorithm was used to define 429 (5%) as “highly allergic”; this group was referred to receive immunization under medical supervision. A total of 98% of the highly allergic individuals had no allergic reaction, 6 (1%) had mild allergic responses, and 3 (0.7%) had anaphylactic reactions.

**Meaning:**

This study’s findings suggest that a simple algorithm enables immunization of most patients with a history of allergy, while only patients defined as highly allergic should receive vaccination under medical supervision.

## Teaser

This cohort study describes the assessment and immunization of highly allergic individuals with the Pfizer-BioNTech COVID-19 vaccine, as well as the rate of adverse reactions to the vaccine in this population.

## Introduction

The COVID-19 pandemic has medical, economic, and many other global consequences. Immunization of most of the population seems to be the main tool to prevent morbidity and mortality and to facilitate a return to normalcy. In December 2020, the US Food and Drug Administration (FDA) issued an Emergency Use Authorization for the Pfizer-BioNTech (BNT162b2) COVID-19 vaccine and the Moderna COVID-19 vaccine (mRNA-1273), each administered as 2 doses separated by 21 and 28 days, respectively.^1,2^ Both vaccines are composed of a nanoparticle-encapsulated lipid, nucleoside-modified messenger RNA (mRNA) that encodes the SARS-CoV-2 spike glycoprotein. Both vaccines were found to be approximately 95% effective in preventing COVID-19. Adverse events included mostly pain at the injection site, fatigue, and headache.^3,4^ Nonetheless, several obstacles have occurred on the path of this global immunization program, one of which is the risk of allergic reactions to these vaccines.^5,6,7^

In the clinical trials of the BNT162b2 and Moderna COVID-19 vaccines, participants with a history of an allergic reaction to any component of the vaccine or an allergy to other vaccines were excluded. Furthermore, in the BNT162b2 studies, individuals with other allergies were also excluded from participating.^3,8^ After the FDA Emergency Use Authorization the BNT162b2 and Moderna COVID-19 vaccines for immunization of the general population, anaphylaxis, a severe, rapid-onset multisystem allergic reaction, was reported in 11.1 cases per 1 million injections with the BNT162b2 vaccine.^5,9^ This resulted in an estimated higher risk of anaphylaxis with the BNT162b2 vaccine of up to 10-fold compared with commonly used vaccines.^10^ Since these early estimates were generated, millions more doses of the COVID-19 vaccines were administered, with an updated reported anaphylaxis rate of 4.7 cases per 1 million doses.^7^ Of note, 90% of anaphylactic reactions were among females and 81% reported prior allergies. Although the allergen within the vaccine is yet to be determined, it has been suggested that the polyethylene glycol (PEG) used to construct the nanoparticle-encapsulated lipid of this vaccine is a possible candidate.^10^ The increasing concerns among patients and physicians about allergic reactions to the COVID-19 vaccines led the Centers for Disease Control and Prevention (CDC) and other authorities to publish different recommendations regarding the safety of these vaccines for patients with a history of allergic reactions.^11^ Despite these recommendations, uncertainty remains, particularly among patients with a history of anaphylaxis and/or multiple allergies.

Israel was among the first countries to implement a national COVID-19 immunization program using the BNT162b2 vaccine, beginning December 19, 2020. At the same time, Sheba Medical Center in Ramat Gan, Israel, began receiving thousands of medical requests regarding vaccine safety. Moreover, many individuals had their vaccination deferred by their physicians and/or immunization teams because of “allergic concerns.” Hence, a referral center was opened in the Sheba Medical Center to provide information and rapid assessment of risk of allergy to the BNT162b2 vaccine. We further offered an alternative for vaccination of high-risk individuals under special observation, with the aim to enable immunization for all. In this study, we describe our experience in the assessment and immunization of highly allergic individuals, as well as the rate of adverse reactions to the BNT162b2 vaccine in this population.

## Methods

The COVID-19 vaccine referral center was created in Sheba Medical Center primarily as an information center for vaccine recipients and health care workers. In the vaccine center, a team of trained personnel from the infectious diseases unit and the infection prevention and control unit received applications for vaccination by phone or email. Among their assignments was to define patients as low risk vs high risk for allergies or with contraindication to be immunized according to the Israel Ministry of Health (MOH) and the CDC.^11,12^ Patients could approach the center on their own behalf or via request of their general practitioner (GP). This cohort study followed the Strengthening the Reporting of Observational Studies in Epidemiology (STROBE) reporting guideline for observational studies^13^ and was performed in accordance with the Declaration of Helsinki^14^ and in agreement with the institutional regulations and approval of the Sheba Medical Center institutional review board. Patient consent was waived as the data were deidentified.

Patients at low risk of allergic reactions included those with a history of sensitivity to aeroallergens or insect bite (including patients receiving immunotherapy for these allergens), food, latex, or contrast media or prior nonanaphylactic reaction to a single drug group or those who had chronic urticaria. Patients receiving immunotherapy or biologic therapy were instructed to delay that treatment for at least 1 week after immunization with the BNT162b2 vaccine. Patients at low risk of allergic reactions were recommended to be immunized in regular settings, with 30 minutes of observation after immunization. Patients and their GPs were instructed to inform our referral center of anaphylaxis or any adverse reaction to the vaccine. Moreover, any significant allergic reaction required notification to the MOH in Israel.

Patients who were not clearly at low risk of allergic reactions were instructed to complete a detailed questionnaire and were referred for further assessment at the clinical immunology and allergy department. The questionnaire included 5 questions about: (1) prior allergic or anaphylactic reactions to 1 or more oral or injectable drugs or vaccines, (2) other allergies (eg, insect bites, food, inhaled allergens, or asthma), (3) treatments used during prior allergic reactions (ie, antihistamines and/or glucocorticoids and/or adrenaline, or hospitalization), (4) current use of drugs (ie, adrenaline syringe carriage and/or antihistamine and/or asthma therapy used regularly), and (5) other immune comorbidities (eg, chronic urticaria, mast cell disorders).

Multiple drug allergy was identified if hypersensitivity to more than 1 drug group was reported (eg, penicillin and nonsteroidal anti-inflammatory drugs). Specifically, respiratory disease exacerbated by nonsteroidal anti-inflammatory drugs and aspirin was considered hypersensitivity to a single drug group. Multiple allergies were noted if patients had 2 or more of the following: (1) drug allergy regardless of the number of compounds, (2) insect sting allergy, (3) food allergy, and (4) allergic rhinitis and/or asthma.

Patients were considered to be at high risk for allergic reactions if one of the following was present: (1) prior anaphylactic reaction to any drug or vaccine, (2) multiple drug allergies, (3) multiple allergies, or (4) mast cell disorders, as were patients who were deferred by their GP or local allergist or the immunization team from vaccination in the regular setting because of concerns about allergic reactions. This high-risk group was referred to be immunized with 2 hours of observation by a dedicated allergy team after vaccination. Premedication was not recommended prior to receiving the first dose of the vaccine unless patients were regularly treated with these drugs (eg, antihistamines); in that case, they were instructed to continue their therapy. Patients with an allergy to PEG and/or 2 or more injectable drugs were rejected from vaccination according to the Israeli MOH recommendations at that time.^12^

The second dose of the vaccine was provided in the same setting, and patients were asked to complete an adverse reactions form regarding the 21 days since receiving the first dose of the vaccine. Early adverse reactions were defined as those that occurred within minutes to 2 hours after vaccination and resolved within 24 hours, while late reactions were defined as those that lasted for more than 24 hours. Data were compared between younger recipients (aged 16-55 years) and older vaccine recipients (aged >55 years).

Statistical analysis was performed using SPSS statistics software for Windows, version 27 (IBM Corp). The Fisher exact test and/or χ^2^ tests were used as appropriate. All *P* values were from 2-sided tests and results were deemed statistically significant at *P* < .05.

## Results

During the study period of approximately 8 weeks, 8102 individuals sent vaccine-related questions to the infectious diseases and infection prevention and control team; 6883 individuals (85.0%) were recommended to receive immunization in the regular settings. The other 1219 individuals (15.0%) were required to complete a questionnaire and were referred for assessment by the clinical immunology and allergy team. After physician assessment of all questionnaires, an additional 785 individuals were defined as being at low risk and were referred to receive immunization in the regular settings. No serious allergic reactions were reported back to our referral center by patients or their GP after immunization in the regular settings.

**Figure 1.**
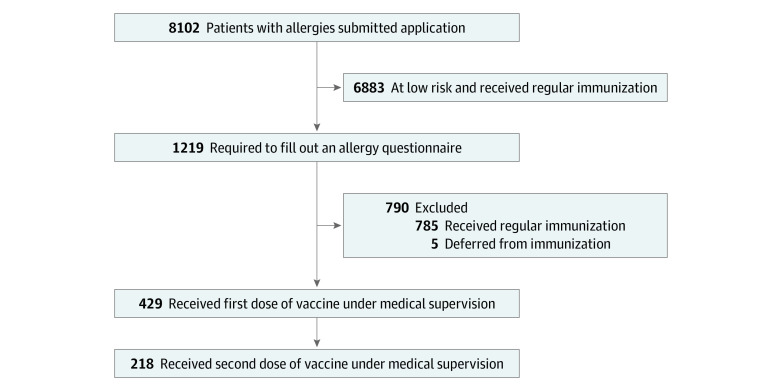
Algorithm of Allergy Assessment Applications The diagram represents the implementation of the allergy risk assessment algorithm among 8102 allergic patients who applied to the COVID-19 referral center from December 27, 2020, to February 22, 2021. Regular immunization indicates immunization in a manner similar to the general population.

Five patients (0.1%) were found to be ineligible for immunization by the clinical and immunology team according to the Israeli MOH instructions (2 patients owing to known sensitivity to PEG and 3 patients with multiple anaphylactic reactions to different injectable drugs). A relatively small group of 429 patients (5.3%) (304 women) were defined as highly allergic and recommended to receive immunization under special observation (Figure 1 and Table). Of these 429 patients, 304 (70.9%) were women, and they had a mean (SD) age of 52 (16) years. This group of highly allergic patients had an extensive history of allergic reactions, namely, prior anaphylaxis (271 [63.2%]), multiple allergies (130 [30.3%]), multiple drug allergies (141 [32.9%]), carriers of adrenaline syringe (95 [22.1%]), food allergy (68 [15.9%]), and regular use of antihistamines (62 [14.5%]) (Table).

**Table.** Demographic Characteristics and History of Highly Allergic Patients

Characteristic	Participants, No. (%) (N = 429)
Age, mean (SD), y	52 (16)
Female	304 (70.9)
Male	125 (29.1)
Prior allergies	429 (100)
Drug allergy	258 (60.1)
Injectable drug allergy	118 (27.5)
Insect bite allergy	60 (14.0)
Food allergy	134 (31.2)
Allergic rhinitis	160 (37.3)
Asthma	96 (22.4)
Multiple allergies^a^	130 (30.3)
Prior anaphylaxis (total)	271 (63.2)
Drugs	141 (32.9)
Insect bite	59 (13.8)
Food	68 (15.9)
Multiple anaphylaxis	49 (11.4)
Adrenalin syringe carriers	95 (22.1)
Permanent antihistamine use	62 (14.5)

^a^ Multiple allergies were defined by 2 or more of the following: (1) drug allergy regardless of number of compounds, (2) insect sting allergy, (3) food allergy, and (4) allergic rhinitis and/or asthma.

### Early Reactions After the First Dose of Vaccine

During the 2 hours of observation after vaccination, 9 women (2.1%) experienced allergic reactions. Mild immediate allergic reactions occurred in 6 patients (1.4%) and included skin eruption or flushing, swelling of the tongue or uvula, or cough that resolved with antihistamine treatment during the observation period. Anaphylactic reactions were documented in 3 patients (0.7%), appeared within 10 to 20 minutes after vaccination, and included significant bronchospasm, skin eruption, itching, and shortness of breath in all 3 patients; angioedema in 2 patients; and gastrointestinal symptoms in 1 patient. They were treated with adrenaline, antihistamines, and an inhaled bronchodilator; 1 patient also received systemic glucocorticoids. Symptoms resolved within 2 to 6 hours and no patients required hospitalization. Two of these patients had a prior diagnosis of multiple drug allergies, and 1 patient had food allergy with anaphylaxis and asthma. These 3 patients did not receive the second dose of the vaccine and did not receive any other COVID-19 vaccine because no other vaccines were available in Israel during the study period. All 9 patients who experienced an immediate reaction to the first dose were followed up by our team within 2 weeks; none reported recurrent or ongoing allergic symptoms. Nonallergic adverse events occurred in 10 patients (2.3%) and included dizziness (3 patients), paresthesia (5), and vagal reactions (2). Most vaccinated highly allergic individuals (412 [96.0%]) did not develop any immediate adverse event.

### Late Adverse Events After the First Dose of Vaccine

**Figure 2.**
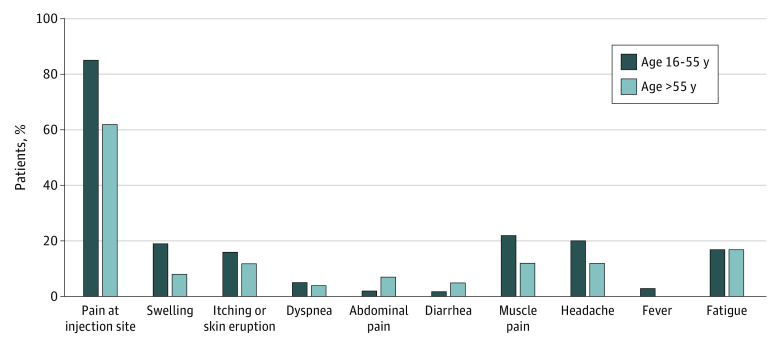
Local and Systemic Reactions Reported After the First Dose of the Pfizer BioNTech COVID-19 Vaccine According to Age Statistically significant differences between the 2 age groups were found for pain at injection site (*P* < .001) and swelling (*P* = .02). Local reactions were pain at injection site and swelling; the rest are systemic reactions.

Local and systemic adverse events, either allergic or nonallergic, were documented prior to the second dose of immunization (ie, ≤21 days after the first dose) (Figure 2). As of February 22, 2021, 218 highly allergic patients (50.8%) received the second dose. Comparable to the Pfizer-BioNTech phase 3 clinical trial,^3^ the most common adverse event was pain at injection site, which was less frequent among participants older than 55 years than among younger participants (108 of 127 [85.0%] vs 56 of 91 [61.5%]; *P* < .001). The most common systemic adverse events were fatigue, headache, and muscle pain in both age groups. Appearance of skin eruption, itching, or urticaria in the days after the first dose was 14.7% (32 of 218) in this highly allergic cohort.

### Early Reactions After the Second Dose of Vaccine

As of February 22, 2021, 218 highly allergic patients also received the second dose of the BNT162b2 vaccine (ie, 21 days after the first dose). During observation, 214 patients (98.2%) had no allergic reactions, whereas 4 patients (1.8%) had minor allergic reactions: 3 patients developed flushing and 1 patient developed flushing and cough that responded to treatment with antihistamines and bronchodilators. Of these 4 patients, 3 also had mild reactions after the first dose and received premedication (eg, antihistamine and bronchodilators) prior to receiving the second dose, whereas 1 patient had a reaction only after the second dose.

## Discussion

A nationwide immunization rollout with the BNT162b2 vaccine was initiated in Israel in December 2020. As of March 4, 2021, 4 847 286 people had received the first dose, and more than 3.5 million of these people had received 2 doses of the BNT162b2 vaccine.^15^ Concerns regarding safety and, particularly, severe allergic reactions to this vaccine are a significant obstacle to implementation of this large immunization program. To our knowledge, this is the first study to report on a special immunization program with the BNT162b2 vaccine for highly allergic patients. In this study, we created an algorithm for rapid screening of individuals who were potentially at risk of immediate allergic reaction to the BNT162b2 vaccine. This simple method can be easily implemented in any country and allowed 95% of applicants to be immunized in the regular settings in Israel.

In our cohort, 429 patients (5.3%) were defined as being at high risk for an allergic reaction and were referred to receive immunization under medical observation. In this group, the mean age was 52 years, similar to the cohort in the Pfizer-BioNTech phase 3 clinical trial.^3^ The Pfizer-BioNTech phase 3 trial comprised 49.4% women, whereas our cohort included 70.9% women. This finding could be expected, as the overall trend of allergic diseases, and particularly drug allergies, are seen more often in women than men.^16,17^ Among our highly allergic adult patients, few risk factors may be suggested: history of multiple drug allergies, drug-related anaphylaxis, and/or multiple allergies. In our cohort, food allergy was reported by 68 patients (15.9%) and multiple drug allergies by were reported by 141 patients (32.9%), whereas in the general adult population less than 5% has a food allergy^18^ and less than 1% has severe or multiple drug allergies.^19^ Moreover, 95 patients (22.1%) in our cohort reported carrying an adrenaline syringe, which may explain why some were deferred by their GP or the immunization teams from vaccination in regular settings.

In this study, we vaccinated this highly allergic group in a special setting that provided reassurance to both patients and physicians and enabled immunization. Nonetheless, in this high-risk group, 2% of patients experienced an allergic reaction either to the first or second dose of the vaccine, of which 3 reactions (0.7%) were anaphylaxis to the first dose. Allergic reactions to vaccines are either acute or late in onset.^20^ Acute-onset reactions are mediated by preformed IgE antibodies against a vaccine component or via other mechanisms that induce immediate mast cell activation. These reactions occur within minutes to several hours after vaccination.^21^ Vaccine-associated anaphylaxis is rare, with an estimated incidence of 1 reaction per 1 million injections for most known vaccines.^22^ This rate was reported to be higher for the BNT162b2 vaccine in the CDC’s reports, indicating 4.7 to 11.1 anaphylaxis cases per 1 million doses, of which 81% were related to a history of allergies or anaphylactic reactions.^5^ More recently, Blumenthal et al^23^ prospectively studied Mass General Brigham employees who received their first dose of mRNA COVID-19 vaccine (either BNT162b2 or Moderna) and reported an even higher anaphylaxis rate. More specifically, in the Mass General Brigham cohort, the rate of allergic reactions to the BNT162b2 vaccine was 1.95% and the rate of anaphylactic reactions was 0.025%.^5,6,7,10^ This relatively higher rate in the Mass General Brigham cohort was explained by the investigators as a concomitant allergy history that was reported by most vaccine recipients who experienced anaphylaxis, while 31% had experienced prior anaphylactic reactions. Similarly, in our cohort allergic reactions to the BNT162b2 vaccine were observed in 1.4% after the first dose and 1.8% after the second dose and anaphylactic reactions were observed in 0.7% only after the first dose. In our highly allergic cohort, prior anaphylaxis was reported by 63% of patients. This finding suggests that, although the precise risk factors for allergic reactions to the BNT162b2 vaccine are yet to be revealed, prior high-risk allergies may enable screening of patients at risk for allergic response to this vaccine. Nonetheless, most patients in our cohort were safely immunized and all allergic and anaphylactic reactions were treated successfully at the immunization site with no requirement for hospitalization and/or further intervention. Furthermore, most high-risk patients who did not experience any adverse events from the first dose also did not exhibit a reaction to the second dose. This finding may suggest that a shortened observation period may be sufficient after the second dose of the vaccine for patients who did not experience a reaction to the first dose. Late adverse events in our cohort were relatively comparable with those previously reported (except for skin eruption and itching, which were present in 15% of this group of allergic patients and not otherwise reported).

### Limitations

Our study has several limitations. The history of allergic reactions was provided by patients’ reports and medical records, many of which were evaluated by allergists through the years. However, no assessment was performed prior to vaccination. In addition, it would have been useful to assess the time since the last allergic reaction or when adrenaline was last used. However, in a real-life setting, based on patient’s recall, these data may be biased and therefore were not included in our analysis.

Data on most patients who were immunized in regular settings can only be estimated, as those patients were not actively followed up. Nonetheless, to our knowledge, among the group of patients defined as being at low risk, no severe allergic reactions were reported to our center or to the MOH in Israel. Although few low-risk patients reported minimal reactions, this lower rate could not be ascertained and thus was not included in our analysis. Owing to the relatively small number of total allergic reactions in our study, we could not assess links between factors that defined the entire highly allergic group and the actual reaction to the vaccine. More studies are needed to define the exact risk factors for anaphylaxis among this highly allergic group. The mechanisms of reactions (eg, tryptase levels or sensitivity to the vaccine ingredients [eg, PEG 2000]), were not measured, as these tests are not performed in our medical center or are currently not available. Finally, late reactions were reported by patients with no other medical documentation of the reactions.

## Conclusions

The rate of allergic reactions, particularly anaphylactic reactions, to the BNT162b2 vaccine are higher than for other commonly used vaccines. According to our study as well as previous reports, this finding is particularly true for patients with a history of allergic reactions. However, this vaccine prevents deadly disease and is a main tool to control the COVID-19 pandemic. Thus, immunization of the general population, including those with an allergic history, is an important goal. Overcoming safety concerns and predominantly allergic ones is needed to achieve this goal. In this study, we enabled immunization of most paitents with allergies by using a simple algorithm that included a referral center, a risk assessment questionnaire, and a safe environment for immunization of highly allergic patients with observation after immunization. This algorithm can be implemented in any medical setting to allow immunization for all.
